# A Novel GBSM for Non-Stationary V2V Channels Allowing 3D Velocity Variations

**DOI:** 10.3390/s21093271

**Published:** 2021-05-10

**Authors:** Naeem Ahmed, Boyu Hua, Qiuming Zhu, Kai Mao, Junwei Bao

**Affiliations:** The Key Laboratory of Dynamic Cognitive System of Electromagnetic Spectrum Space, College of Electronic and Information Engineering, Nanjing University of Aeronautics and Astronautics, Nanjing 211106, China; jubayar123@nuaa.edu.cn (N.A.); byhua@nuaa.edu.cn (B.H.); maokai@nuaa.edu.cn (K.M.); broadenway@nuaa.edu.cn (J.B.)

**Keywords:** V2V channel, NS GBSM, 3D velocity variations, 3D scattering environments, statistical property

## Abstract

A new non-stationary (NS) geometry-based stochastic model (GBSM) is presented for developing and testing the communication systems of vehicle-to-vehicle (V2V) applications, which considers the three-dimensional (3D) scattering environments and allows 3D velocity as well. In this paper, the proposed GBSM for NS V2V channels allowed 3D velocity variations and was more suitable for actual V2V communications because it provided smoother transitions between the consecutive channel segments. The time-variant channel coefficient and the channel parameters, i.e., Doppler frequencies, path delay and power, angle of arrival (AoA), and angle of departure (AoD), were analyzed and derived. Likewise, the theoretical statistical properties as the probability density function (PDF), the auto-correlation function (ACF), and Doppler power spectral density (DPSD) were also analyzed and derived under the von Mises–Fisher (VMF) distribution. Finally, the theoretical and measured results were well coordinated alongside the implemented results, which confirmed the feasibility of the introduced model along with the theoretical expressions.

## 1. Introduction

Vehicle-to-vehicle (V2V) communication is the most important development of the modern intelligent transportation systems (ITSs), which are more accurate and realistic. As a result of sharing information among the vehicles, infrastructures, and other devices, it has gained an enormous interest lately because of its great ability to sustain intelligent transportation [1,2]. In recent years, V2V communication systems have shown key potentiality with the help of the fifth-generation (5G) and beyond 5G (B5G), along with the future sixth-generation (6G) technologies [3,4]. Because of the traffic efficiency and security-relevant cases, V2V communication helps to improve the safety of lives and resources by sharing information in real time under sophisticated transportation [5,6,7,8]. However, the ability and credibility of V2V communications are impacted by crowded space, vehicles, movements of scatterers, etc. [9,10]. Furthermore, the motions of both terminals and scatterers result in the time-variant feature of the platform, which differentiates V2V channels from conventional fixed-to-mobile (F2M) channels [11,12,13]. Thus, a realistic, precise, and easy channel model is needed.

### 1.1. Related Works

Due to the mild precision and difficulty, the GBSM is one key form of the V2V channel model. Since the assumption of wide-sense stationary (WSS) is just for a brief amount of time, several non-WSS V2V GBSMs were proposed successively during the past [14,15,16]. Two-dimensional (2D) and 3D NS V2V GBSMs were analyzed with static clusters or moving scatterers in [17,18,19,20]. The vehicles, pedestrians, and infrastructures can disperse or shift in the 3D plane under real-life scenarios [21]. Recently, a hemisphere [22], two cylinders [23,24], two spheres [24], and several 3D non-stationary V2V channel models were proposed by expanding the scatterers’ allocation on the plane of the 3D regulated form. In contrast to the theoretical results in [25,26], the outcome of phases linked to the Doppler frequencies of [22,23,24] were improper. Therefore, an updated 3D channel model was addressed in [27] with accurate Doppler frequency. By taking notice of the fact that the majority of the mentioned models took only the fixed velocity of the vehicle into account, in realistic traffic environments, the velocity could be time-variant, whereas the time-variant velocities were taken into account in [28,29,30,31], where the trajectories were 2D and also 1D for simplicity. Very recently, a non-stationary GBSM among the terminal and vehicle with 3D arbitrary trajectories and 3D antenna arrays with the consideration of fixed velocity was presented in [32].

### 1.2. Main Contributions

The concept of this paper was obtained from [31,32] to model the V2V channels allowing 3D trajectories, but the proposed model only supported 3D constant velocity. The goal of this paper was to address this void. The key achievements and novelties are defined as follows:

(1) Considering the idea of GBSM and the twin cluster proposal, a new 3D V2V channel model is introduced. In this proposed model, non-stationary V2V channels with the movement of scatterers for non-line of sight (NLoS) scattering scenarios were taken into consideration. Furthermore, each of the terminals allows 3D velocity variations according to the 3D scattering condition, which ensures that the model is much more usual and has the prospect of non-stationarity.

(2) The time-evolving channel variable algorithms for 3D V2V communication scenarios, i.e., Doppler frequencies, AOA and AOD, path delays, and powers, were executed and facilitated.

(3) The hypothetical terms of PDF, ACF, and DPSD of this introduced model according to the VMF scattering scenarios were examined and approved by the assumed and measured outcomes.

This article is arranged as follows. Section 2 introduces the GBSM for V2V channels. Section 3 considers the proposed V2V channel model allowing 3D velocity variations. In Section 4, the time-variant channel parameter computation is described. In Section 5, the theoretical PDFs, ACFs, and DPSDs of this introduced model are executed in detail. The simulated outcomes are compared with the theoretical ones, and the calculated outcomes can be seen and contrasted in Section 6. The conclusions are eventually drawn in Section 7.

## 2. A GBSM for Non-Stationary V2V Channels

**Figure 1:**
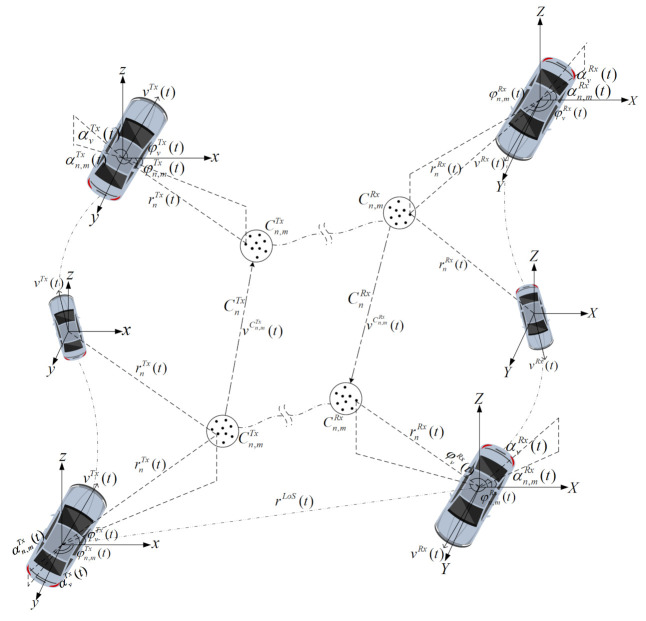
3D GBSM-based V2V communication channel model.

Let us consider a typical NS V2V communication scenario in Figure 1 including the transmitter (Tx), receiver (Rx), and clusters, which is categorized by 3D random trajectories and 3D antenna arrays, where the local coordinate systems of the Tx and Rx are xyz and XYZ, respectively. It can be presumed that the direction of transport or the distance of the Tx or Rx at the initial time corresponds to their own x-axis. The velocity of the Tx or Rx, which is shifting over time, can be denoted by $v^{i}\left(t\right)$ where i∈ {Tx, Rx}. Multiple propagation pathways are used in the channel among each pair of antenna parts, and each route often includes many clusters. The V2V channel propagation consists of various propagation paths like the line of sight (LoS), single bounce, double bounce, and multiple-bounce paths. In addition, the single and double bounce can be considered as special forms of the NLoS. The first and last clusters are denoted under general NLoS scenarios as $C_{n,m}^{Tx}$ and $C_{n,m}^{Rx}$, including time-variant velocities, respectively.

Based on the twin cluster idea, a virtual link derives the 3D location of the first cluster $C_{n,m}^{Tx}$ or the last cluster $C_{n,m}^{Rx}$ and defines an analogous path delay and power. Thus, the periodical diversion of the complex channel impulse response (CIR) for NLoS paths, as well as the LoS path can be represented as:

(1) $$h\left(t,\tau \right)=\sum_{n=1}^{N(t)}\sqrt{P_{n}\left(t\right)}h_{n}\left(t\right)\delta \left(\tau -\tau_{n}\left(t\right)\right)$$

where N(t) refers to the number of directions that can be described by path power $P_{n}\left(t\right)$, channel fading coefficient $h_{n}\left(t\right)$, and path delay $\tau_{n}\left(t\right)$. Through the usual scattering scenarios, the channel fading coefficient in this paper is modeled as:

(2) $$h_{n}\left(t\right)=\lim_{M\to \infty }\;\frac{1}{\sqrt{M}}\sum_{m=1}^{M}e^{j(2\pi \int_{0}^{t}f_{n,m}\left(t^{\prime }\right)dt^{\prime }+\theta_{n,m})}$$

where *M*, $\theta_{n,m}$ represents the numbers of sub-paths and the random phase, respectively, and $f_{n,m}\left(t\right)$ describes the Doppler frequency and can be represented as:

(3) $$\begin{array}{cc} f_{n,m}\left(t\right) & =f_{n,m}^{Tx,C_{n,m}^{Tx}}\left(t\right)+f_{n,m}^{C_{n,m}^{Rx},Rx}\left(t\right)\\ \; & =\frac{1}{\lambda }\left(v^{Tx,C_{n,m}^{Tx}}\left(t\right)\cdot s_{n,m}^{Tx}\left(t\right)+v^{C_{n,m}^{Rx},Rx}\left(t\right)\cdot s_{n,m}^{Rx}\left(t\right)\right)=\frac{1}{\lambda }\sum_{i}^{Tx,Rx}\left(v^{i,C_{n,m}^{i}}\left(t\right)\cdot s_{n,m}^{i}\left(t\right)\right) \end{array}.$$

The relative velocity vector among the vehicles and clusters is represented by $v^{i,C_{n,m}^{i}}\left(t\right)$ where i∈ {Tx, Rx}, and $\lambda =c/f_{0}\;$ is the wavelength, where *c* and $f_{0}$ represent the speed of light and the carrier frequency, respectively. In (3), $s_{n,m}^{i}\left(t\right)$ indicates the unit vectors of the AoD and AoA of the mth sub-path, where i∈ {Tx, Rx}.

(4) $$s_{n,m}^{i}\left(t\right)=\left[\begin{array}{c} \cos\alpha_{n,m}^{i}\left(t\right)\cos\varphi_{n,m}^{i}\left(t\right)\\ \cos\alpha_{n,m}^{i}\left(t\right)\sin\varphi_{n,m}^{i}\left(t\right)\\ \sin\alpha_{n,m}^{i}\left(t\right) \end{array}\right]$$

where $\alpha_{n,m}^{i}\left(t\right)$ and $\varphi_{n,m}^{i}\left(t\right)$ represent the elevation and azimuth angle, respectively.

## 3. Proposed V2V Channel Model

The basic GBSM is suitable for V2V channels, but it is worth mentioning that all channel parameters need to be calculated in real time. To make this model more applicable to real-world V2V communications, the effects of the velocity or trajectory need to be considered. In this paper, we approximated the velocity with the form of the momentum as:

(5) $$v^{i,C_{n,m}^{i}}\left(t\right)=v^{i,C_{n,m}^{i}}\left(0\right)+\int_{0}^{t}a^{i,C_{n,m}^{i}}\left(t^{\prime }\right)dt^{\prime }$$

where $v^{i,C_{n,m}^{i}}\left(0\right)$ denotes the initial relative speed vector and $a^{i,C_{n,m}^{i}}\left(t\right)$ denotes the corresponding speed acceleration vector, respectively. For the 3D time-variant speed acceleration, we can obtain:

(6) $$\begin{array}{cc} \; & \left[\begin{array}{ccc} v_{x}^{i,C_{n,m}^{i}}\left(t\right) & v_{y}^{i,C_{n,m}^{i}}\left(t\right) & v_{z}^{i,C_{n,m}^{i}}\left(t\right) \end{array}\right]\\ \; & =\left[\begin{array}{ccc} v_{x}^{i,C_{n,m}^{i}}\left(0\right) & v_{y}^{i,C_{n,m}^{i}}\left(0\right) & v_{z}^{i,C_{n,m}^{i}}\left(0\right) \end{array}\right]+\int_{0}^{t}\left[\begin{array}{ccc} a_{x}^{i,C_{n,m}^{i}}\left(t^{\prime }\right) & a_{y}^{i,C_{n,m}^{i}}\left(t^{\prime }\right) & a_{z}^{i,C_{n,m}^{i}}\left(t^{\prime }\right) \end{array}\right]dt^{\prime }. \end{array}$$

By taking into account the 3D relative velocity speed and acceleration, both the relative speed and acceleration correspond to the xyz axis and can be rewritten as:

(7) $${∥v∥}^{i,C_{n,m}^{i}}\left(t\right)={∥v∥}^{i,C_{n,m}^{i}}\left(0\right)+\int_{0}^{t}{∥a∥}^{i,C_{n,m}^{i}}\left(t^{\prime }\right)dt^{\prime }$$

(8) $$q_{v}^{i,C_{n,m}^{i}}\left(t\right)=q_{v}^{i,C_{n,m}^{i}}\left(0\right)+\int_{0}^{t}b^{i,C_{n,m}^{i}}\left(t^{\prime }\right)dt^{\prime }$$

where $q_{v}^{i,C_{n}^{i}}\left(0\right)$ and $b^{i,C_{n}^{i}}\left(t\right)$ denote the initial relative movement direction and the acceleration of the direction, respectively. Under the consideration of moving scatterers, the Doppler frequency can be described as [27]:

(9) $$f_{n,m}\left(t\right)=f_{n,m}^{Tx}\left(t\right)+f_{n,m}^{C_{n}^{Tx}}\left(t\right)+\cdots +f_{n,m}^{C_{n}^{Rx}}\left(t\right)+f_{n,m}^{Rx}\left(t\right)$$

where $f_{n,m}^{Tx}\left(t\right)$, $f_{n,m}^{Rx}\left(t\right)$, $f_{n,m}^{C_{n}^{Tx}}\left(t\right),f_{n,m}^{C_{n}^{Rx}}\left(t\right)$ mean the Doppler frequencies due the movements of terminals and scatterers, respectively. Since the scatterers of the virtual link are unknown and have a random movement, it is reasonable to assume that the mean value of the Doppler frequency is zero, which is caused by the virtual scatterers. Moreover, (9) can be simplified as:

(10) $$f_{n,m}\left(t\right)=\frac{1}{\lambda }\left({∥v∥}^{Tx,C_{n,m}^{Tx}}\left(t\right)\cdot q_{v}^{Tx,C_{n,m}^{Tx}}\left(t\right)\cdot s_{n,m}^{Tx}\left(t\right)+{∥v∥}^{C_{n,m}^{Rx},Rx}\left(t\right)\cdot q_{v}^{C_{n,m}^{Rx},Rx}\left(t\right)\cdot s_{n,m}^{Rx}\left(t\right)\right)$$

where ${∥v∥}^{Tx,C_{n,m}^{Tx}}\left(t\right)$ indicates the correlative velocity of the Tx and cluster $C_{n,m}^{Tx}$ and ${∥v∥}^{C_{n,m}^{Rx},Rx}\left(t\right)$ defines the speed of the Rx and cluster $C_{n,m}^{Rx}$, respectively. For the velocities that are changing, the movement direction vectors can be defined as:

(11) $$\begin{array}{cc} \; & q_{v}^{Tx,C_{n,m}^{Tx}}\left(t\right)=q_{v}^{Tx,C_{n,m}^{Tx}}\left(0\right)+\int_{0}^{t}b^{Tx,C_{n,m}^{Tx}}\left(t^{\prime }\right)dt^{\prime }=\left[\begin{array}{c} \cos\alpha_{v}^{Tx,C_{n,m}^{Tx}}\left(t\right)\cos\varphi_{v}^{Tx,C_{n,m}^{Tx}}\left(t\right)\\ \cos\alpha_{v}^{Tx,C_{n,m}^{Tx}}\left(t\right)\sin\varphi_{v}^{Tx,C_{n,m}^{Tx}}\left(t\right)\\ \sin\alpha_{v}^{Tx,C_{n,m}^{Tx}}\left(t\right) \end{array}\right]\\ \; & q_{v}^{C_{n,m}^{Tx},Rx}\left(t\right)=q_{v}^{C_{n,m}^{Rx},Rx}\left(0\right)+\int_{0}^{t}b^{C_{n,m}^{Rx},Rx}\left(t^{\prime }\right)dt^{\prime }=\left[\begin{array}{c} \cos\alpha_{v}^{C_{n,m}^{Rx},Rx}\left(t\right)\cos\varphi_{v}^{C_{n,m}^{Rx},Rx}\left(t\right)\\ \cos\alpha_{v}^{C_{n,m}^{Rx},Rx}\left(t\right)\sin\varphi_{v}^{C_{n,m}^{Rx},Rx}\left(t\right)\\ \sin\alpha_{v}^{C_{n,m}^{Rx},Rx}\left(t\right) \end{array}\right] \end{array}.$$

In (11), $q_{v}^{i,C_{n,m}^{i}}\left(t\right)$, $\varphi_{v}^{i,C_{n,m}^{i}}\left(t\right)$, $\alpha_{v}^{i,C_{n,m}^{i}}\left(t\right)$ represents the direction vector, azimuth angle, and elevation angle, respectively, where i∈ {Tx, Rx}. Combining (10) and (11) with the help of [33], the Doppler frequency for NLoS path $f_{n,m}\left(t\right)$ can be written as:

(12) $$\begin{array}{cc} f_{n,m}\left(t\right) & =\frac{1}{\lambda }\left[\begin{array}{cc} \; & {∥v∥}^{Tx,C_{n,m}^{Tx}}\left(t\right)\left[\begin{array}{c} \cos\alpha_{v}^{Tx,C_{n,m}^{Tx}}\left(t\right)\cos\varphi_{v}^{Tx,C_{n,m}^{Tx}}\left(t\right)\\ \cos\alpha_{v}^{Tx,C_{n,m}^{Tx}}\left(t\right)\sin\varphi_{v}^{Tx,C_{n,m}^{Tx}}\left(t\right)\\ \sin\alpha_{v}^{Tx,C_{n,m}^{Tx}}\left(t\right) \end{array}\right]\cdot \left[\begin{array}{c} \cos\alpha_{n,m}^{Tx}\left(t\right)\cos\varphi_{n,m}^{Tx}\left(t\right)\\ \cos\alpha_{n,m}^{Tx}\left(t\right)\sin\varphi_{n,m}^{Tx}\left(t\right)\\ \sin\alpha_{n,m}^{Tx}\left(t\right) \end{array}\right]+\\ \; & {∥v∥}^{C_{n,m}^{Rx},Rx}\left(t\right)\left[\begin{array}{c} \cos\alpha_{v}^{C_{n,m}^{Rx},Rx}\left(t\right)\cos\varphi_{v}^{C_{n,m}^{Rx},Rx}\left(t\right)\\ \cos\alpha_{v}^{C_{n,m}^{Rx},Rx}\left(t\right)\sin\varphi_{v}^{C_{n,m}^{Rx},Rx}\left(t\right)\\ \sin\alpha_{v}^{C_{n,m}^{Rx},Rx}\left(t\right) \end{array}\right]\cdot \left[\begin{array}{c} \cos\alpha_{n,m}^{Rx}\left(t\right)\cos\varphi_{n,m}^{Rx}\left(t\right)\\ \cos\alpha_{n,m}^{Rx}\left(t\right)\sin\varphi_{n,m}^{Rx}\left(t\right)\\ \sin\alpha_{n,m}^{Rx}\left(t\right) \end{array}\right] \end{array}\right]\\ \; & =\frac{1}{\lambda }\left[\begin{array}{cc} \; & \left({∥v∥}^{Tx,C_{n,m}^{Tx}}\left(0\right)+\int_{0}^{t}{∥a∥}^{Tx,C_{n,m}^{Tx}}\left(t^{\prime }\right)dt^{\prime }\right)\\ \; & \left(\begin{array}{cc} \; & \cos\left(\varphi_{n,m}^{Tx}\left(t\right)-\varphi_{v}^{Tx,C_{n,m}^{Tx}}\left(t\right)\right)\cos\alpha_{n,m}^{Tx}\left(t\right)\cos\alpha_{v}^{Tx,C_{n,m}^{Tx}}\left(t\right)+\\ \; & \sin\alpha_{n,m}^{Tx}\left(t\right)\sin\alpha_{v}^{Tx,C_{n,m}^{Tx}}\left(t\right) \end{array}\right)+\\ \; & \left({∥v∥}^{C_{n,m}^{Rx},Rx}\left(0\right)+\int_{0}^{t}{∥a∥}^{C_{n,m}^{Rx},Rx}\left(t^{\prime }\right)dt^{\prime }\right)\\ \; & \left(\begin{array}{cc} \; & \cos\left(\varphi_{n,m}^{Rx}\left(t\right)-\varphi_{v}^{C_{n,m}^{Rx},Rx}\left(t\right)\right)\cos\alpha_{n,m}^{Rx}\left(t\right)\cos\alpha_{v}^{C_{n,m}^{Rx},Rx}\left(t\right)+\\ \; & \sin\alpha_{n,m}^{Rx}\left(t\right)\sin\alpha_{v}^{C_{n,m}^{Rx},Rx}\left(t\right) \end{array}\right) \end{array}\right]. \end{array}$$

Hence, the transceivers and the scatterers are moving, so the calculation of the Doppler frequency is complicated; for the sake of simplicity, the overall Doppler frequency of this proposed model can be represented as (12). The movement of transceivers and scatterers comes into consideration in (12) where the mathematical term of the AoAs, AoDs, and the velocity vectors of the Tx, Rx, and clusters are also shown. By substituting (12) into (2), we can obtain the channel fading coefficient, as well as the parametric V2V channel model with respect to the 3D velocity variations.

## 4. Time-Variant Parameter Computations

### 4.1. Time-Variant Distances

During the movement of transceivers Tx, Rx, and clusters $C_{n}^{Tx}$, $C_{n}^{Rx}$, the location vector for a very short time interval of the transceivers or the clusters can be represented as:

(13) $${∥r∥}_{n}^{i}\left(t\right)={∥r∥}_{n}^{i}\left(0\right)+\int_{0}^{t}\left(\left({∥v∥}_{n}^{i}\left(0\right)\right)+\int_{0}^{\tau }\left({∥a∥}_{n}^{i}\left(t^{\prime }\right)\right)dt^{\prime }\right)d\tau$$

(14) $${∥r∥}_{n}^{C_{n}^{i}}\left(t\right)={∥r∥}_{n}^{C_{n}^{i}}\left(0\right)+\int_{0}^{t}\left(\left({∥v∥}_{n}^{C_{n}^{i}}\left(0\right)\right)+\int_{0}^{\tau }\left({∥a∥}_{n}^{C_{n}^{i}}\left(t^{\prime }\right)\right)dt^{\prime }\right)d\tau$$

where ${∥r∥}_{n}^{i}\left(t\right)$ and ${∥r∥}_{n}^{C_{n}^{i}}\left(t\right)$ denote the 3D relative location of the transceivers and clusters on x-, y-, and z-axes, respectively. Due to the mobility of the clusters and terminals, the distance between the Tx and $C_{n}^{Tx}$ or between the Rx and $C_{n}^{Rx}$ can be represented by ${∥D∥}_{n}^{i}\left(t\right)$, where i∈ {Tx, Rx}, which is based on the correlative movements. In this paper, it can be expressed as:

(15) $${∥D∥}_{n}^{i}\left(t\right)=\sqrt{{\left(∥r_{n,x}^{C_{n}^{i}}\left(t\right)-r_{n,x}^{i}\left(t\right)∥\right)}^{2}+{\left(∥r_{n,y}^{C_{n}^{i}}\left(t\right)-r_{n,y}^{i}\left(t\right)∥\right)}^{2}+{\left(∥r_{n,z}^{C_{n}^{i}}\left(t\right)-r_{n,z}^{i}\left(t\right)∥\right)}^{2}}.$$

According to the location vector of the Tx and $C_{n}^{Tx}$ or the Rx and $C_{n}^{Rx}$, the instantaneous location of both the transceivers and clusters can be represented as (16) and (17), respectively, for the x-, y-, and z-axes:

(16) $$\begin{array}{cc} \; & r_{n,x}^{i}\left(t\right)=\cos\left(\varphi_{n}^{i}\left(t\right)-\varphi_{v}^{C_{n}^{i}}\right)\cos\alpha_{n}^{i}\left(t\right)\\ \; & r_{n,y}^{i}\left(t\right)=\cos\left(\varphi_{n}^{i}\left(t\right)-\varphi_{v}^{C_{n}^{i}}\right)\sin\alpha_{n}^{i}\left(t\right)\\ \; & r_{n,z}^{i}\left(t\right)=\sin\alpha_{n}^{i}\left(t\right) \end{array}$$

(17) $$\begin{array}{cc} \; & r_{n,x}^{C_{n}^{i}}\left(t\right)=\cos\left(\varphi_{n}^{C_{n}^{i}}\left(t\right)-\varphi_{v}^{i}\right)\cos\alpha_{n}^{C_{n}^{i}}\left(t\right)\\ \; & r_{n,y}^{C_{n}^{i}}\left(t\right)=\cos\left(\varphi_{n}^{C_{n}^{i}}\left(t\right)-\varphi_{v}^{i}\right)\sin\alpha_{n}^{C_{n}^{i}}\left(t\right)\\ \; & r_{n,z}^{C_{n}^{i}}\left(t\right)=\sin\alpha_{n}^{C_{n}^{i}}\left(t\right) \end{array}.$$

### 4.2. Time-Variant AoAs and AoDs

By considering the displacements of the terminals and clusters, the AoAs and AoDs excessively vary with time. Both the azimuth and elevation angles can be represented by the random parameters φ and α and can be characterized by a definite PDF. A small number of preceding works already proposed that under various scenarios, the AoA and AoD may follow the Gaussian uniform distribution. Nevertheless, the measured and analyzed outcomes in [28,32] showed that the VMF distribution is responsive and can describe such angle allocations. Therefore, in this paper, it was presumed that the AoA and AoD carry out the following distribution as:

(18) $$p\left(\alpha ,\varphi \right)=\frac{\cos\alpha \exp\left(\kappa^{i}\left(\cos\left(\varphi^{i}-{\bar{\varphi }}^{i}\right)\cos\alpha^{i}\cos{\bar{\alpha }}^{i}+\sin\alpha^{i}\sin{\bar{\alpha }}^{i}\right)\right)}{{\left(2\pi \right)}^{m/2}I_{m/2-1}\left(\kappa \right)\left(\kappa^{1-m/2}\right)}$$

where $\kappa^{1-m/2}$ represents the element correlated with the distribution concentration, $I_{m/2-1}\left(\cdot \right)$ denotes the zeroth-order modified Bessel function of the first kind, and ${\bar{\varphi }}^{i}\left(t\right)$, ${\bar{\alpha }}^{i}\left(t\right)$ represent the marginal value of the AoAs and AoDs, respectively. Moreover, in the order m/2−1, the factor *m* represents the 3D and 2D VMF distribution, while m=3 and m=2, respectively. The value of the distribution factor κ remains constant, while the simulation time is very short. Therefore, when κ=0, this means a special case of the isotropic scattering scenario. For the time-variant AoAs and AoDs, the mean angles are also time variant and can be denoted by ${\bar{\varphi }}^{i}\left(t\right)$ and ${\bar{\alpha }}^{i}\left(t\right)$ under non-stationary scenarios, while κ can be obtained by measurement. Accordingly, we can obtain the geometrical relationship as:

(19) $${\bar{\varphi }}_{n}^{i}\left(t\right)=\left\{\begin{array}{cc} \; & \arccos\left(\frac{r_{n,x}^{C_{n}^{i}}\left(t\right)-r_{n,x}^{i}\left(t\right)}{{∥D∥}_{n}^{i}\left(t\right)}\right),r_{n,y}^{C_{n}^{i}}\left(t\right)-r_{n,y}^{i}\left(t\right)\geq 0\\ \; & -\arccos\left(\frac{r_{n,x}^{C_{n}^{i}}\left(t\right)-r_{n,x}^{i}\left(t\right)}{{∥D∥}_{n}^{i}\left(t\right)}\right),r_{n,y}^{C_{n}^{i}}\left(t\right)-r_{n,y}^{i}\left(t\right)<0 \end{array}\right.$$

and:

(20) $${\bar{\alpha }}_{n}^{i}\left(t\right)=\arcsin\left(\frac{r_{n,z}^{C_{n}^{i}}\left(t\right)-r_{n,z}^{i}\left(t\right)}{{∥D∥}_{n}^{i}\left(t\right)}\right)$$

where $\left[r_{n,x}^{C_{n}^{i}}\left(t\right),r_{n,y}^{C_{n}^{i}}\left(t\right),r_{n,z}^{C_{n}^{i}}\left(t\right)\right]$ and $\left[r_{n,x}^{i}\left(t\right),r_{n,y}^{i}\left(t\right),r_{n,z}^{i}\left(t\right)\right]$ represent the location vectors of the x-, y-, and z-axes, respectively, and ${∥D∥}_{n}^{i}\left(t\right)$ denotes the distance over time.

### 4.3. Time-Variant Path Delays and Powers

The total delay at a moment of time *t* involves the first bounce $\tau_{n}^{Tx}\left(t\right)$, the virtual link ${\tilde{\tau }}_{n}\left(t\right)$, and the last bounce $\tau_{n}^{Rx}\left(t\right)$, which is obtained by:

(21) $$\tau_{n}\left(t\right)=\frac{∥r^{Tx}\left(t\right)-r^{C_{n}^{Tx}}\left(t\right)∥+∥r^{Rx}\left(t\right)-r^{C_{n}^{Rx}}\left(t\right)∥}{c}+{\tilde{\tau }}_{n}\left(t\right)$$

where ${\tilde{\tau }}_{n}\left(t\right)$ represents the corresponding delay of the virtual link. For multipath scenarios, the propagation power from the transmitter to the receiver varies with different path delays, which depends on the real environment. Based on the measurement results in the 3GPP channel model, the path power with respect to the delay can be calculated by:

(22) $$P_{n}^{\prime }\left(t\right)=\exp\left\{-\tau_{n}\left(t\right)\frac{\eta_{\tau }-1}{\eta_{\tau }\sigma_{\tau }}10^{-\frac{\vartheta_{n}}{10}}\right\}$$

where $\eta_{\tau }$, $\sigma_{\tau }$ denote the delay distribution and delay spread and $\vartheta_{n}$ denotes the shadow term, which follows the Gaussian distribution. Note that the total power should be normalized for all the paths. Moreover, the total power at the moment of time *t* includes the power of subsisted clusters from the preceding moment of time and the power of recently produced clusters.

## 5. Statistical Properties for the Proposed Model

### 5.1. Time-Variant PDF

The characteristic function of a random variable $h_{i,n}$ can be written as [34]:

(23) $$\psi_{h_{i,n}}\left(t,x\right)=\int_{-\infty }^{\infty }p_{h_{i,n}}\left(t,z\right)e^{j2\pi zx}dz=J_{0}\left(2\pi c_{i,n}x\right)$$

where *x* is a real-valued variable, i=1,2,⋯, and $n=1,2,\cdots N_{i}$. The PDF of the sum of statistically significant random variables is often estimated by using the characteristic function. By combining random variables’ characteristic functions, it yields:

(24) $$\psi_{h_{i,n}}\left(t,x\right)=\psi_{h_{1,n}}\left(t,x\right)\cdot \psi_{h_{2,n}}\left(t,x\right)\cdots \psi_{h_{i,n}}\left(t,x\right)=\prod_{n=1}^{N_{i}}J_{0}\left(2\pi c_{i,n}x\right).$$

By the help of the characteristic functions, the PDF for the first random variable can be defined as:

(25) $$p_{h_{1,n}}\left(t,z_{1}\right)=\int_{-\infty }^{\infty }\psi_{h_{1,n}}\left(t,x\right)e^{-j2\pi zx}dx=2\int_{0}^{\infty }\prod_{n=1}^{N_{1}}\left(2\pi xc_{1,n}\right)\cos\left(2\pi xz\right)dx$$

where $p_{h_{1,n}}\left(t,z\right)$ represents the PDF of the first random variable *z*. Thus, both $p_{h_{1,n}}\left(t,z_{1}\right),p_{h_{2,n}}\left(t,z_{2}\right)$ are statistically independent for all $n=1,2,\cdots N_{i}$. Therefore, the joint probability density function of the random variables is represented as:

(26) $$p_{h_{1,n}}p_{h_{2,n}}\left(t,z_{1},z_{2}\right)=p_{h_{1,n}}\left(t,z_{1}\right)\cdot p_{h_{2,n}}\left(t,z_{2}\right)=p_{h_{1,n}}\left(t,z_{1}\right)\cdot \delta \left(z_{2}-g\left(t,z_{1}\right)\right)$$

where δ and *g* define the functions of the random variable and can be described as:

(27) $$g\left(t,z_{1}\right)=\left\{\begin{array}{c} c_{n}\sqrt{1-{\left(z_{1}/c_{n}\right)}^{2}},\left|z_{1}\right|\leq c_{n}\\ 0,\left|z_{1}\right|>c_{n} \end{array}\right.$$

where $c_{n}$, $z_{1}$ denotes the Doppler coefficient and random variable, respectively. After transforming the Cartesian coordinates to polar coordinates for two random variables as $z_{1}=z\cos\theta$ and $z_{2}=z\sin\theta$, with the help of Equations (25) and (26), we can obtain the envelope PDF for NLoS scenarios as:

(28) $$p_{h}\left(t,z\right)={\left(2\pi \right)}^{2}z\int_{0}^{\infty }\left[\prod_{n=1}^{N_{i}}J_{0}\left(2\pi c_{n}x\right)\right]J_{0}\left(2\pi zx\right)xdx$$

where $c_{n}=P_{n}\left(t\right)/\sqrt{N_{i}}\;$ and θ is over −π to π. In (28), it is defined that $p_{h}\left(t,z\right)$ can be explained completely by the parameters $N_{i}$ and path power $P_{n}\left(t\right)$, whereas the frequency parameters do not have any effect. Since within every time period, $P_{n}\left(t\right)$ is approximately unchanged, the output PDF is determined by $N_{i}$. It is worth mentioning that the envelope PDF of an SOC model is the approximation of the Rayleigh PDF while $N_{i}\to \infty$. Therefore, it can easily be proven that (28) tends to be the Rayleigh distribution.

### 5.2. Time-Variant ACF

For non-stationary channels, the time-variant ACF at different time instants turns into a function of the time delay τ and time *t*. It can be measured between random variables, which increases the predictability of random events. For continuous time-signals, the ACF characterizes the time correlation of a stochastic process. The ACF under non-stationary scattering scenarios is a time-variant function of time delay τ and time *t*. The time-variant ACF can be specified as:

(29) $$r_{hh}\left(t,\tau \right)\;=\;E\left\{h^{*}\left(t\right)h\left(t\;+\;\tau \right)\right\}$$

where ${\left(\cdot \right)}^{*}$ defines the complex conjugation operation. The computation of (29) defines that the ACF shifts through the time delay. The time-variant ACF can be specified as:

(30) $$r_{hh}\left(t,\tau \right)=\int_{-\pi }^{\pi }\int_{-\pi }^{\pi }\int_{-\pi }^{\pi }\int_{-\pi }^{\pi }\begin{array}{c} \sqrt{p\left(\varphi_{n}^{Tx}\left(t+\tau \right),\alpha_{n}^{Tx}\left(t+\tau \right)\right)}\sqrt{p\left(\varphi_{n}^{Tx}\left(t\right),\alpha_{n}^{Tx}\left(t\right)\right)}\\ \sqrt{p\left(\varphi_{n}^{Rx}\left(t+\tau \right),\alpha_{n}^{Rx}\left(t+\tau \right)\right)}\sqrt{p\left(\varphi_{n}^{Rx}\left(t\right),\alpha_{n}^{Rx}\left(t\right)\right)}\\ e^{-j2\pi \int_{t}^{t+\tau }f_{n,m}\left(t^{\prime }\right)dt^{\prime }}d\varphi_{n}^{Tx}d\alpha_{n}^{Tx}d\varphi_{n}^{Rx}d\alpha_{n}^{Rx} \end{array}$$

(31) $$r_{hh}\left(t,\tau \right)=\int_{-\pi }^{\pi }\int_{-\pi }^{\pi }\sqrt{p\left(\varphi_{n}^{i}\left(t+\tau \right),\alpha_{n}^{i}\left(t+\tau \right)\right)}\sqrt{p\left(\varphi_{n}^{i}\left(t\right),\alpha_{n}^{i}\left(t\right)\right)}e^{-j2\pi \int_{t}^{t+\tau }f_{n,m}\left(t^{\prime }\right)dt^{\prime }}d\varphi_{n}^{i}d\alpha_{n}^{i}$$

where $i\in \left\{Tx,Rx\right\}$ when the clusters $C_{n}^{Tx}$ and $C_{n}^{Rx}$ are independent of each other, and (31) is equal to the product of the ACFs at Tx and Rx. For simplicity, the antenna array is positioned at the root of the coordinate system. With the help of (10), we can simplify (31) as:

(32) $$\begin{array}{cc} \; & {\tilde{r}}_{hh}\left(t,\tau \right)=\frac{1}{{\left(4\pi \right)}^{m}I_{m/2-1}^{2}\left(\kappa \right)\left(\kappa^{1-m}\right)}\\ \; & \int_{-\pi }^{\pi }\int_{-\pi }^{\pi }\left(\begin{array}{cc} \; & \sqrt{\exp\kappa^{i}\left(\cos\alpha \left(\begin{array}{cc} \; & \cos\left(\varphi^{i}\left(t+\tau \right)-{\bar{\varphi }}_{n}^{i}\left(t+\tau \right)\right)\cos\alpha^{i}\left(t+\tau \right)\\ \; & \cos{\bar{\alpha }}_{n}^{i}\left(t+\tau \right)+\sin\alpha^{i}\left(t+\tau \right)\sin{\bar{\alpha }}_{n}^{i}\left(t+\tau \right) \end{array}\right)\right)}\\ \; & \cdot \sqrt{\exp\kappa^{i}\left(\cos\alpha \left(\begin{array}{cc} \; & \cos\left(\varphi^{i}\left(t\right)-{\bar{\varphi }}_{n}^{i}\left(t\right)\right)\cos\alpha^{i}\left(t\right)\\ \; & \cos{\bar{\alpha }}_{n}^{i}\left(t\right)+\sin\alpha^{i}\left(t\right)\sin{\bar{\alpha }}_{n}^{i}\left(t\right) \end{array}\right)\right)}\\ \; & \cdot e^{-j2\pi \int_{t}^{t+\tau }{∥v∥}^{i,C_{n}^{i}}\left(t^{\prime }\right)\cdot q_{v}^{i,C_{n}^{i}}\left(t^{\prime }\right)\cdot s_{n,m}^{i}\left(t^{\prime }\right)} \end{array}\right)d\varphi_{n}^{i}d\alpha_{n}^{i} \end{array}.$$

In (32), the form of the distribution remains fixed, and the relative angle offset is nearly fixed at a very short time interval when $\kappa^{i}$ is constant or nearly constant over a short time interval, for a few milliseconds. Here, (32) can be described as:

(33) $$\begin{array}{cc} \; & {\tilde{r}}_{hh}\left(t,\tau \right)=\frac{1}{{\left(4\pi \right)}^{m}I_{m/2-1}^{2}\left(\kappa \right)\left(\kappa^{1-m}\right)}\\ \; & \int_{-\pi }^{\pi }\int_{-\pi }^{\pi }\left(\begin{array}{cc} \; & \sqrt{\exp\kappa^{i}\left(\cos\alpha \left(\begin{array}{cc} \; & \cos\left(\varphi^{i}\left(t+\tau \right)-{\bar{\varphi }}_{n}^{i}\left(t+\tau \right)\right)\cos\alpha^{i}\left(t+\tau \right)\\ \; & \cos{\bar{\alpha }}_{n}^{i}\left(t+\tau \right)+\sin\alpha^{i}\left(t+\tau \right)\sin{\bar{\alpha }}_{n}^{i}\left(t+\tau \right) \end{array}\right)\right)}\\ \; & \cdot \sqrt{\exp\kappa^{i}\left(\cos\alpha \left(\begin{array}{cc} \; & \cos\left(\varphi^{i}\left(t\right)-{\bar{\varphi }}_{n}^{i}\left(t\right)\right)\cos\alpha^{i}\left(t\right)\\ \; & \cos{\bar{\alpha }}_{n}^{i}\left(t\right)+\sin\alpha^{i}\left(t\right)\sin{\bar{\alpha }}_{n}^{i}\left(t\right) \end{array}\right)\right)}\cdot e^{-j2\pi \int_{t}^{t+\tau }A\cdot B} \end{array}\right)d\varphi_{n}^{i}d\alpha_{n}^{i} \end{array}$$

where:

(34) $$\begin{array}{cc} \; & A=\left({∥v∥}^{i,C_{n}^{i}}\left(0\right)+\int_{0}^{t}{∥a∥}^{i,C_{n}^{i}}\left(t^{\prime }\right)dt^{\prime }\right)\\ \; & B=\left(\cos\left(\varphi_{n}^{i}\left(t\right)-\varphi^{i,C_{n}^{i}}\left(t\right)\right)\cos\alpha_{n}^{i}\left(t\right)\cos\alpha^{i,C_{n}^{i}}\left(t\right)+\sin\alpha_{n}^{i}\left(t\right)\sin\alpha^{i,C_{n}^{i}}\left(t\right)\right) \end{array}.$$

The ACFs mentioned in (33) are more common and even appropriate for several propagation scenarios. Finally, by submitting the values of (34) into (33), we can obtain the approximate expression of this proposed model.

### 5.3. Time-Variant DPSD

The Fourier transform of ACF $r_{hh}\left(t,\tau \right)$ is called the DPSD, which denotes a continuous spectrum of the Doppler frequency. It describes how the power of a signal or time series is distributed over the frequency. The time-variant DPSD of the proposed model can be obtained as:

(35) $$S_{hh}\left(f,t\right)\;=\;\int_{-\infty }^{\infty }r_{hh}\left(t,\tau \right)\exp\left\{-j2\pi f\tau \right\}d\tau$$

where time-variant DPSD $S_{hh}\left(f,t\right)\;$ is established by the Fourier transform of $r_{hh}\left(t,\tau \right)$ having a highly consistent trend to the time delay τ.

## 6. Simulation and Analysis of the Results

**Figure 2:**
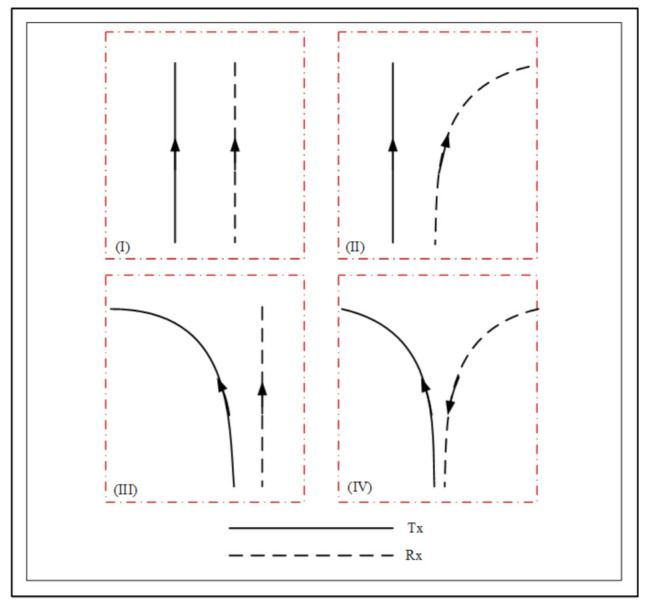
Different trajectories of the Tx and Rx.

This section describes the simulated method focused on the suggested model and verifies the results with the simulated, theoretical, and measured ones. In this simulation, the AoA and AoD are described to follow the VMF distribution with κ=1, and the carrier frequency is 2.48 GHz. We assumed that the shifting of scatterers is evenly divided around two terminals. The arbitrary variables are as follows, $∥v^{C_{n}^{i}}∥\tilde{\;}U\left(0,1\right)$ m/s, where *U* denotes the uniform distribution, $\varphi_{v}^{C_{n}^{Tx}}$, $\varphi_{v}^{C_{n}^{Rx}}$, and $\alpha_{v}^{C_{n}^{Tx}}$, $\alpha_{v}^{C_{n}^{Rx}}$ are evenly allocated over $\left(-\pi ,\pi \right)$. In addition, the Tx and Rx shift over various trajectories, and the specific velocity parameters can be found as follows:

To verify the simulation results with the theoretical results, four different trajectories, which are related to realistic V2V communication scenarios, were considered. Figure 2 shows four different trajectories of the Tx and Rx as the same direction, straight-right turn, left-straight turn, and left-right turn in the opposite direction, respectively. All the variables, i.e., the 3D relative velocity speed ${∥v∥}^{i}$, the acceleration ${∥a∥}^{i}$, and the angle variables $\alpha_{v}^{i}$, affect the amplitude and varying patterns of the channel statistical characteristics.

**Figure 3:**
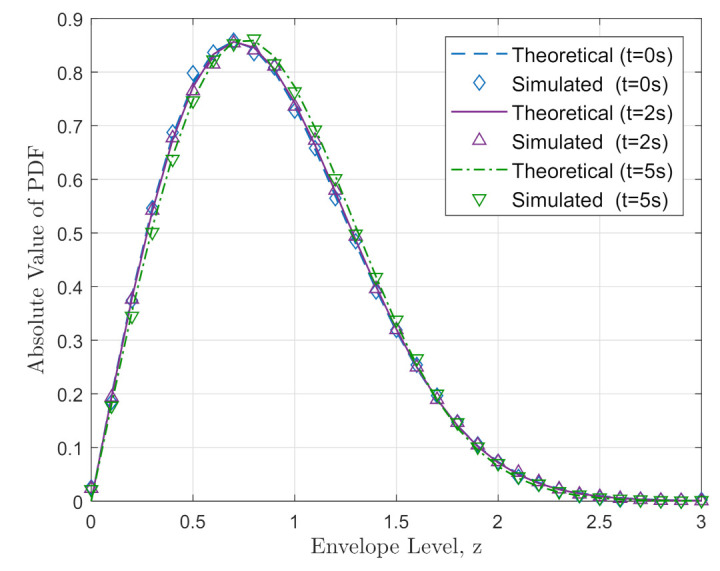
Theoretical and simulated PDF at different time instants.

In this paper, we considered the envelope PDFs, ACFs, and DPSDs for a few moments of time in order to verify the simulated output along with the theoretical output. Therefore, in (28), the PDF result of this introduced model at three different moments of time where t= 0 s, 2 s, and 5 s differentiates along the analytical output in Figure 3. Because of the time-variant channel parameters, the simulated PDFs change with the corresponding theoretical PDFs. The channel parameters in (28) indicate the variation of time through the simulation process, which is random. The simulated PDF of Figure 3 clarifies the Rayleigh channel fading where all the intervals are considered to have normalized power. The figure shows a good similarity of the simulated PDF with the theoretical Rayleigh PDF.

**Figure 4:**
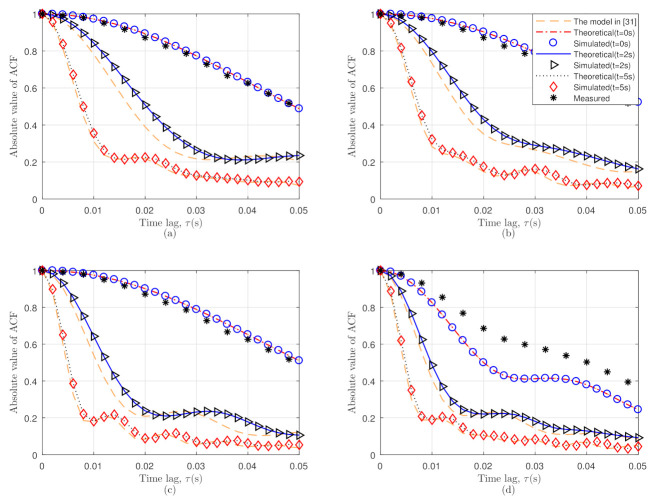
Theoretical and simulated ACF at different time instants.

By comparing the theoretical and calculated outcomes of the ACFs, the proposed model was validated by considering four distinct standard V2V scenarios with trajectory variations of the Tx and Rx. All of the scenarios in Figure 2 were considered at various speeds with initial directions. The theoretical and simulated ACFs at three different moments were derived and shown in Figure 4. By comparing the ACFs of this model and the reference model in [31] at various time instants, it was obvious that the velocity parameters had a significant impact on the ACFs. Due to the fact that the model in [31] only considered 2D velocity variations, the proposed model provided better simulated outcomes with respect to the theoretical outcomes when the velocity changed arbitrarily. The measurement result from [27] was considered, which came into consideration to compare with this proposed model, and likewise, the measurement result of [27] was also considered in this paper. Figure 4a–d demonstrates the theoretical and simulated ACFs of our proposed model at various time instants under four different scenarios using (29)–(34). Through the comparison of Figure 4a,b the ACFs under straight-right turn changed faster than in the same direction. Besides that, in Figure 4c,d it is easy to obtain that the left-right opposite turn changed faster than the left-straight turn for the different initial values of the channel parameters. As the ACFs were shifting at a faster rate, it was fair to assume that the larger the parameter value, the more complex the communication scenarios would be and that the ACFs would change faster. The simulation results of the PDFs and ACFs of this method were consistent with the theoretical results. Thorough the different moments of time, the evaluated outcomes of the PDFs and ACFs showed a good agreement with the relevant theoretical outcomes, which proved that the simulated outcomes were relevant with respect to the theoretical ones.

**Figure 5:**
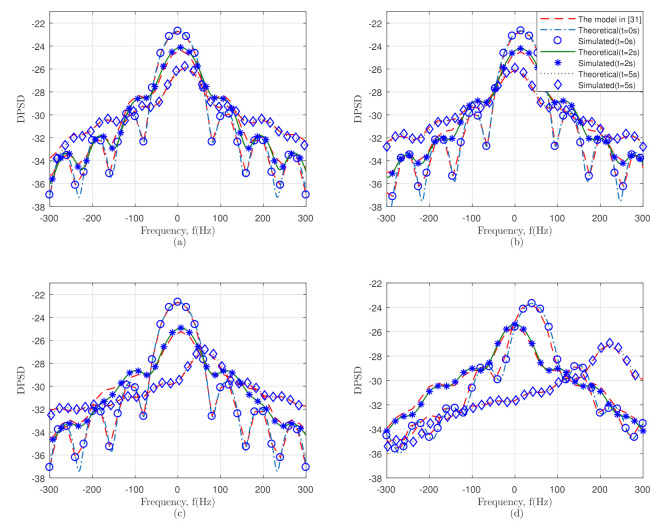
Theoretical and simulated DPSD at different time instants.

According to Table 1, all the simulation parameters are described for every different scenario, i.e., for the Tx and Rx in Scenario I, which have the same direction, where the velocity parameter ${∥v∥}^{i}=0.6,0.8$ (ms${}^{-1}$), the acceleration parameter ${∥a∥}^{i}=0.8,0.6$ (ms${}^{-2}$), and the azimuth and elevation angle $\varphi_{v}^{i}=\pi /2\;,\pi /2\;$ and $\alpha_{v}^{i}=0,0$, respectively. Similarly, for all the other scenarios, the Doppler frequency changed over time accordingly. Besides, the hypothetical and evaluated DPSDs at various moments of time, i.e., t= 0 s, 2 s, and 5 s were analyzed and are derived in Figure 5. The DPSDs of the introduced model and the model in [31] were calculated and are compared in Figure 5. In this paper, we considered the 3D velocity variation for different time instants, while the model in [31] merely considered 2D velocity variations. Thus, the DPSDs of the proposed model were more precise than the model in [31] compared to the theoretical results.

In addition, the velocity parameters $v^{Tx},v^{Rx}$ and the acceleration parameters $a^{Tx},a^{Rx}$ affected the Doppler frequency, which showed the perfect comprehension among the theoretical and simulated consequences over time. Figure 5 also demonstrates the comparison between the reference model [31] and our model, respectively. The observation of the Doppler frequency changed over time where $\varphi_{v}^{i}$ and $\alpha_{v}^{i}$ affected the amplitude of the Doppler frequency. Figure 5 shows that the analytical and evaluated outcomes of the DPSDs of the mentioned scenarios illustrated velocity variation with traveling scatterers, which was a confirmation of the non-isotropic scattering scenarios. It can also be seen that using (12), the channel parameters influenced the Doppler frequency at different moments of time, such as t = 0s, 2s, and 5s. The overall Doppler frequency in Figure 5d is much bigger than that in Figure 5c; similarly, that in Figure 5b is comparatively bigger than that in Figure 5a, which indicates that the Doppler frequency was affected by the initial value of the movement direction. By comparing the Doppler frequencies at various time intervals in various scenarios, the effect of the velocity variations of the Tx and Rx can clearly be seen. Ultimately, the good agreement of the theoretical and experimental outcomes in Figure 5 assures the exactness of the introduced model.

## 7. Conclusions

A novel GBSM for the NS V2V channel method allowing 3D trajectories with velocity variations was presented in this paper. The introduced model was much more compatible for the real V2V communication scenario. All the channel parameters were thoroughly derived and analyzed during the simulation process, which showed the great impact of the velocity variation on the channel characteristics for both the Tx and Rx. We also derived and simulated the PDF, ACF, and DPSD of the introduced model, showing a proper comprehension among the theoretical, measured, and implemented results. The proposed model and the evolving theoretical results are very useful for realistic performance estimation and improvement of V2V communication systems. Moreover, the new model will be useful in the future for developing, analyzing, and testing practical V2V communication systems.

## Figures and Tables

**Table 1:** Velocity parameters of the Tx and Rx.

*Scenarios*	${∥v∥}^{i}$	${∥a∥}^{i}$	$\varphi_{v}^{i}$	$\alpha_{v}^{i}$
*I*	0.6,0.8 (ms${}^{-1}$)	0.8,0.6 (ms${}^{-2}$)	$\frac{\pi }{2},\frac{\pi }{2}$	0,0
*II*	0.5,0.6 (ms${}^{-1}$)	0.8,1 (ms${}^{-2}$)	$\frac{\pi }{2},\frac{\pi }{2}$	$0,\frac{\pi }{12}$
*III*	0.9,1 (ms${}^{-1}$)	1,0.8 (ms${}^{-2}$)	$\frac{\pi }{2},\frac{\pi }{2}$	$\frac{\pi }{18},0$
*IV*	1,0.7 (ms${}^{-1}$)	0.7,1 (ms ${}^{-2}$)	$\frac{\pi }{2},-\frac{\pi }{2}$	$\frac{\pi }{12},\frac{\pi }{18}$

## Data Availability

Not applicable.
